# Extensive bilateral pulmonary embolism revealing iron deficiency—masked JAK2-positive polycythemia vera: A case report

**DOI:** 10.1016/j.radcr.2026.06.035

**Published:** 2026-06-23

**Authors:** Hossam-Eddine Ejjebli, Soumia Boulouiz, Zakaria Bazid, Nabila Ismaili, Noha El Ouafi

**Affiliations:** a Faculty of Medicine and Pharmacy, Mohammed Ist University, Oujda, Morocco; b Department of Cardiology, Mohammed VI University Hospital, Mohammed Ist University, Oujda, Morocco; c Laboratory of Epidemiology, Faculty of Medicine and Pharmacy, Clinical Research and Public Health, Oujda, Morocco

**Keywords:** Polycythemia vera, Masked polycythemia vera, Pulmonary embolism, Iron deficiency, JAK2 V617F mutation, Myeloproliferative neoplasm

## Abstract

Polycythemia vera is a BCR-ABL1–negative myeloproliferative neoplasm associated with increased thrombotic risk. Pulmonary embolism as the initial manifestation of iron deficiency–masked polycythemia vera is uncommon and may be difficult to recognize when hemoglobin and hematocrit are not overtly elevated. We report the case of a 67-year-old postmenopausal woman with no identifiable thromboembolic risk factors who presented with acute dyspnea. Computed tomography pulmonary angiography showed extensive bilateral pulmonary embolism involving proximal and distal pulmonary arterial branches, with a right ventricular-to-left ventricular diameter ratio greater than 1, consistent with right ventricular strain. Transthoracic echocardiography confirmed right ventricular dilation and systolic dysfunction, with a tricuspid annular plane systolic excursion of 9 mm. No deep venous thrombosis was identified. The association of unprovoked pulmonary embolism, right ventricular dysfunction, and subtle hematologic abnormalities prompted evaluation for an underlying myeloproliferative neoplasm. Laboratory tests showed an elevated red blood cell count with high-normal hemoglobin (15 g/dL) and hematocrit (45.9%), marked microcytosis with a mean corpuscular volume of 66.5 fL, and low mean corpuscular hemoglobin of 21.7 pg. Further workup revealed low ferritin, low transferrin saturation, and subnormal serum erythropoietin. Molecular testing identified a JAK2 V617F mutation, and bone marrow biopsy showed hypercellular marrow with trilineage proliferation. The patient received therapeutic anticoagulation, low-dose aspirin, and hydroxyurea. This case highlights the role of multimodal imaging in diagnosis and risk stratification, and the need to consider masked polycythemia vera in unprovoked pulmonary embolism with unexplained microcytosis and elevated red blood cell count.

## Introduction

Polycythemia vera (PV) is one of the classical BCR-ABL1-negative myeloproliferative neoplasms characterized by clonal proliferation of the myeloid lineage, most commonly driven by the JAK2 V617F mutation. It is classically associated with increased red cell mass, often accompanied by leukocytosis and thrombocytosis, and carries a well-established risk of thrombotic complications, which remain a major cause of morbidity and mortality [1]. According to the 2022 International Consensus Classification (ICC) [2], the diagnosis of PV is based on elevated hemoglobin or hematocrit levels (or increased red cell mass), characteristic bone marrow findings with trilineage proliferation, and the presence of JAK2 mutations, with subnormal serum erythropoietin as a minor diagnostic criterion.

A subset of patients with PV does not fulfill the usual hemoglobin or hematocrit thresholds at presentation despite otherwise characteristic features of the disease; these cases are commonly referred to as masked polycythemia vera and may remain clinically occult, resulting in delayed recognition [3].

This diagnostic pitfall becomes particularly relevant when the initial clinical manifestation is thrombotic rather than hematologic. Although both arterial and venous thromboses are well recognized in PV, pulmonary embolism as the presenting manifestation is uncommon [4], particularly when overt erythrocytosis is absent. In this setting, subtle abnormalities such as an elevated red blood cell count, microcytosis, low erythropoietin, or unexplained iron deficiency may provide important clues to an underlying myeloproliferative neoplasm.

We report a case of an extensive bilateral pulmonary embolism revealing iron deficiency–masked JAK2-positive PV, highlighting the diagnostic challenge posed by high-normal hemoglobin and hematocrit levels and emphasizing the importance of considering occult PV in cases of unprovoked venous thromboembolism.

## Case

We report the case of a 67-year-old postmenopausal woman who was admitted to the emergency department for acute-onset dyspnea at rest. Her cardiovascular risk factors included hypertension and type 2 diabetes mellitus. She had no prior history of venous thromboembolism, recent surgery, immobilization, estrogen therapy, active cancer, or other identifiable provoking factors. However, she reported a history of intermittent postmenopausal bleeding, which had not been previously investigated.

On admission, she was hemodynamically stable with a blood pressure of 138/85 mmHg, a heart rate of 100 bpm, and tachypnea. Oxygen saturation was 85% on room air, improving to 95% with 5 L/min supplemental oxygen. She was afebrile at 36.8°C.

Physical examination showed no signs of deep vein thrombosis, jugular venous distension, hepatosplenomegaly, or lymphadenopathy.

A 12-lead electrocardiogram (Fig. 1) showed a regular sinus rhythm (97 beats/min) with an S1Q3T3 pattern and flattened T waves in the inferior leads.

**Fig. 1:**
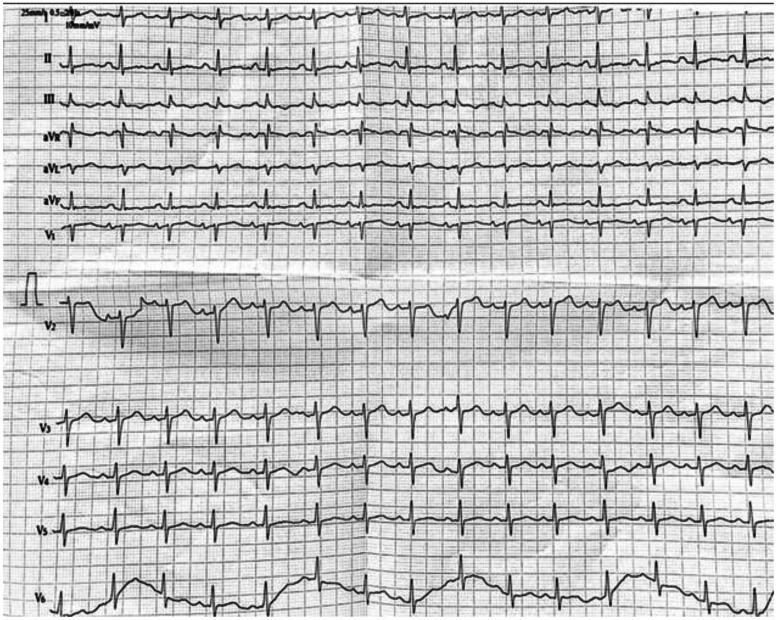
Twelve-lead ECG showing sinus rhythm with S1Q3T3 pattern suggestive of acute right heart strain, supporting urgent cardiopulmonary evaluation and further imaging assessment.

Computed tomography pulmonary angiography (CTPA), performed due to acute dyspnea and elevated D-dimer levels, showed extensive bilateral pulmonary arterial filling defects, involving the left pulmonary artery and multiple lobar, segmental, and subsegmental branches bilaterally. The RV/LV diameter ratio was greater than 1, indicating right ventricular strain. Pulmonary parenchyma assessment revealed no evidence of pulmonary infarction or consolidation, and no features suggestive of chronic thromboembolic disease, such as eccentric or organized thrombi, arterial webs, or mosaic attenuation, were present. No pleural effusion was observed (Fig. 2).

**Fig. 2:**
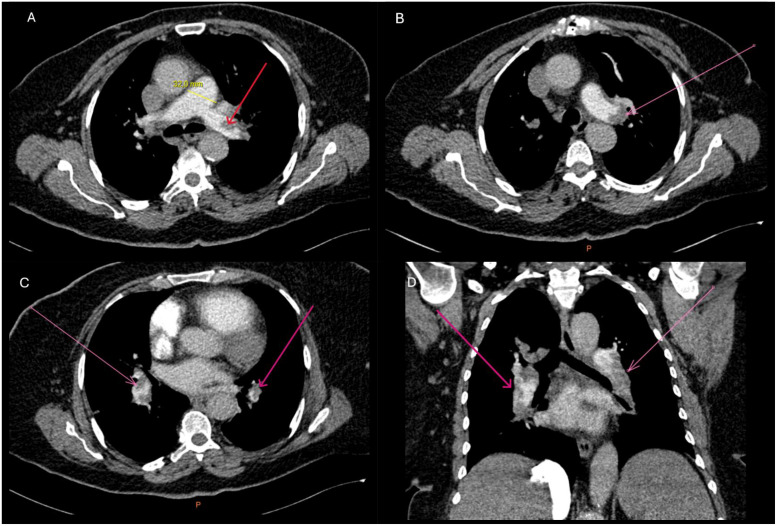
Contrast-enhanced computed tomography pulmonary angiography (CTPA) showing extensive bilateral pulmonary embolism: (A) Axial CTPA image at the level of the pulmonary arterial bifurcation showing enlargement of the pulmonary trunk measuring 32 mm (yellow arrow), with a hypodense intraluminal filling defect within the left pulmonary artery, consistent with thrombus (red arrow). (B) Axial CTPA demonstrating extension of the thrombus into the left lobar and segmental pulmonary arterial branches (pink arrow). (C) Axial CTPA image showing bilateral distal pulmonary emboli involving lobar, segmental and subsegmental branches (pink arrow) and (D) Coronal CT slice showing extensive bilateral pulmonary embolism, involving the left pulmonary and multiple right lobar branches with proximal and distal extension (pink arrow). The extensive thrombotic burden and CT evidence of right ventricular strain, later confirmed by echocardiography, contributed to risk stratification and supported intensive cardiac care unit admission with close monitoring.

Transthoracic echocardiography (Fig. 3) revealed right ventricular dilation (RV/LV ratio 1.0) with systolic dysfunction (TAPSE 9 mm; tricuspid annular S’ wave of 0.07 m/s) and paradoxical septal motion. Pulmonary acceleration time was shortened with a mid-systolic notch on pulmonary flow Doppler, and systolic flow reversal was noted in the hepatic veins. The left ventricle was nondilated and hypertrophic with preserved systolic function (LVEF 57% by Simpson’s biplane). No pericardial effusion was present.

**Fig. 3:**
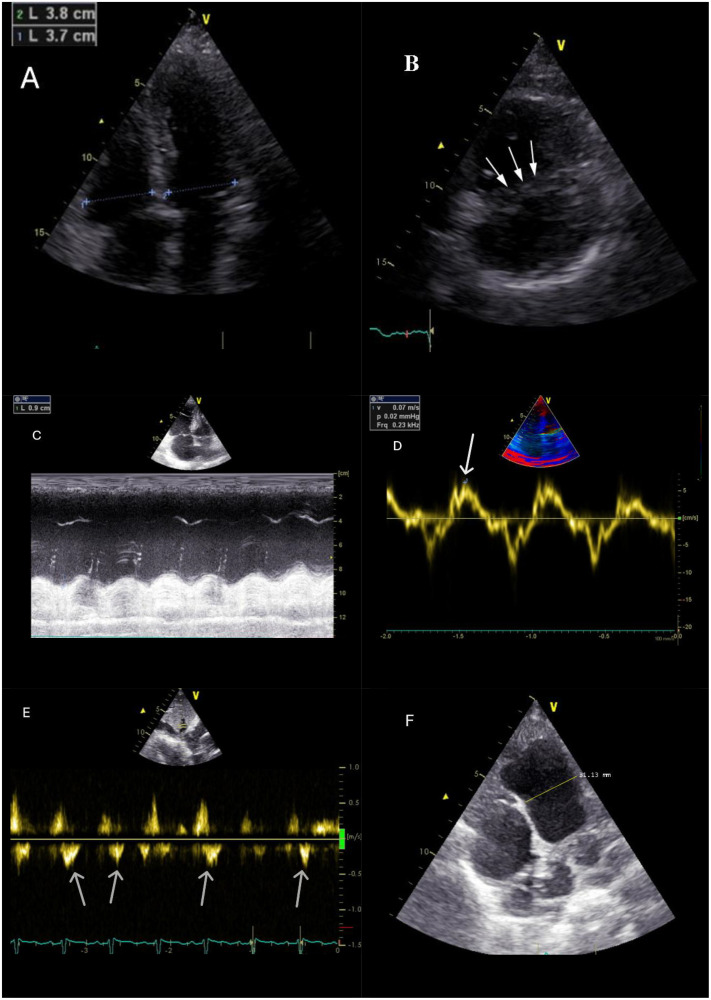
Transthoracic echocardiography showing right ventricular overload: (A) Parasternal long-axis view showing right ventricular dilation with an RV/LV diameter ratio of approximately 1. (B) Parasternal short-axis view showing a D-shaped left ventricle and interventricular septal flattening (white arrows). (C) M-mode showing reduced tricuspid annular plane systolic excursion (TAPSE). (D) Tissue Doppler shows a reduced S’ wave velocity of 0.07 m/s (white arrow). (E) Reversed systolic S wave in the hepatic vein pulsed Doppler (white arrow) and (F) Enlargement of the main pulmonary artery with an end-diastolic diameter of 31.13 mm (yellow line). The echocardiographic findings contributed to intermediate-high-risk pulmonary embolism stratification, supporting intensive cardiac monitoring and therapeutic management.

In the absence of hemodynamic instability, but with right ventricular dysfunction on imaging and elevated cardiac biomarkers, the pulmonary embolism was classified as intermediate-high risk. The sPESI score was 1. Given the patient’s high thrombotic-risk phenotype, therapeutic anticoagulation was initiated with weight-adjusted low-molecular-weight heparin at a therapeutic dose, and the patient was closely monitored in the intensive cardiac care unit. The decision to combine therapeutic anticoagulation with low-dose aspirin was individualized after multidisciplinary assessment, while carefully monitoring bleeding risk. Following stabilization, anticoagulation was transitioned to rivaroxaban 15 mg twice daily for 3 weeks, followed by 20 mg once daily, according to current guidelines.

Initial laboratory tests showed a markedly elevated D-dimer level of 7.75 µg/mL, elevated high-sensitivity troponin (318 ng/L; reference <26 ng/L), elevated NT-proBNP (3831 pg/mL), and an inflammatory response with C-reactive protein of 106 mg/L (reference <5 mg/L). The white blood cell count was 13,170/µL (reference 4000-10,000/µL), with neutrophilia of 9330/µL (reference 1500-7000/µL).

Red blood cell count was elevated at 7.37 × 10^^6^/µL, whereas hemoglobin was 15.0 g/dL (reference for women, 12-16 g/dL) and hematocrit was 45.9% (reference for women, 37%-47%). Marked microcytosis (MCV 66.5 fL) and low mean corpuscular hemoglobin (MCH 21.7 pg) were present, while the platelet count remained within the normal range at 155,000/µL. Iron studies showed a ferritin level of 3 ng/mL (reference 10-125 ng/mL) and transferrin saturation of 10% (reference >30%). There was no biological evidence of hemolysis, with normal lactate dehydrogenase, bilirubin levels, and haptoglobin. The reticulocyte count was mildly elevated at 158,800/µL. The laboratory findings are summarized in Table 1.

**Table 1:** Laboratory investigations at admission and 1-year follow-up.

Parameter	Admission	Follow-up (1 year)	Reference range
Hemoglobin	15.0 g/dL	14.0 g/dL	12-16 g/dL (female)
Hematocrit	45.9%	42.6%	37-47% (female)
Red blood cells	7.37 × 10⁶/µL	4.40 × 10⁶/µL	4.0-5.2 × 10⁶/µL
MCV	66.5 fL	96 fL	80-100 fL
MCH	21.7 pg	31.8 pg	27-33 pg
White blood cells	13,170/µL	8580/µL	4000-10,000/µL
Platelets	155,000/µL	188,000/µL	150,000-400,000/µL
Ferritin	3 ng/mL	33 ng/mL	10-125 ng/mL
Transferrin saturation	10%	40%	>30%
LDH	316 IU/L	-	<480 IU/L
Total bilirubin	6.53 mg/L	-	<10 mg/L
Direct bilirubin	2.91 mg/L	-	<3 mg/L
Haptoglobin	1.240 g/L	-	0.300-2.000 g/L
Reticulocytes	158,800/µL	-	20,000-80,000/µL
Erythropoietin	2.3 mIU/mL	-	2.6-18.5 mIU/mL

LDH, lactate dehydrogenase; MCH, mean corpuscular hemoglobin; MCV, mean corpuscular volume.

An extended etiological work-up was undertaken. Cervico-thoraco-abdomino-pelvic contrast-enhanced CT scan showed no evidence of malignancy, tumor markers were negative, and lower limb venous Doppler ultrasonography showed no deep vein thrombosis. Furthermore, BCR-ABL1 rearrangement was not detected; serum erythropoietin was subnormal at 2.3 mIU/mL (reference 2.6-18.5 mIU/mL). Molecular testing identified a JAK2 V617F mutation with a variant allele frequency of 14.4%.

Bone marrow biopsy showed a hypercellular marrow with panmyelosis involving erythroid, granulocytic and megakaryocytic lineages. Megakaryocytes were pleomorphic, mature forms with loose clustering. Reticulin staining showed no condensation of the reticulin network (MF0), and iron stores were reduced. CD34 immunostaining showed no increase in blast cells or abnormal clusters of immature precursors, with a blast percentage of less than 5%.

Red cell mass measurement was not available at our center. Given the patient’s age and the absence of personal or family history of thrombosis, extensive inherited thrombophilia screening was not performed, as it was considered of limited additional diagnostic value in this context.

Although hemoglobin (15 g/dL) and hematocrit (45.9%) did not exceed conventional diagnostic thresholds, the diagnosis of masked PV was strongly supported by the combination of JAK2 V617F positivity, characteristic bone marrow morphology, subnormal serum erythropoietin, elevated red blood cell count, and iron deficiency, as evidenced by marked microcytosis, low ferritin, and low transferrin saturation. These findings strongly suggest that iron deficiency and microcytosis masked the expected hemoglobin and hematocrit elevation of PV.

A gynecologic evaluation was performed. The cervicovaginal cytology results were normal, and a transvaginal ultrasound revealed a submucosal fibroid without any suspicious endometrial abnormalities. The fibroid was subsequently surgically removed, and short-term follow-up was arranged. Gastrointestinal evaluation was planned in the outpatient follow-up.

The patient denied symptoms suggestive of hyperviscosity, including headache, dizziness, erythromelalgia, aquagenic pruritus, or visual disturbances, and no splenomegaly was detected.

Review of prior laboratory results showed no previously documented erythrocytosis, supporting pulmonary embolism as the initial manifestation leading to PV diagnosis.

She was discharged 6 days after admission following clinical stabilization. At discharge, long-term rivaroxaban and PV-directed therapy, including low-dose aspirin and hydroxyurea, were prescribed. A statin was also initiated because of her high cardiovascular risk profile, and she was referred for hematology follow-up. Therapeutic phlebotomy was deferred to avoid further aggravation of the patient’s iron deficiency. Iron supplementation was not started since the iron deficiency was considered secondary to bleeding from the uterine fibroid, which was later surgically removed. At 1-month follow-up, dyspnea had markedly improved and oxygen saturation was 98% on room air. Echocardiography demonstrated resolution of right ventricular dilation with improved systolic function (TAPSE 19.5 mm; S’ wave of 0.12 m/s). D-dimer decreased to 0.8 µg/mL, troponin normalized (18 ng/L), and hematocrit was controlled at 44.5%.

At 3-month follow-up, she remained clinically stable without recurrent thrombotic events, hydroxyurea was titrated to 500 mg twice daily, and she continued regular hematologic follow-up. At 1-year follow-up, hemoglobin was 14 g/dL and hematocrit 42.6% (Table 1), and the patient remained asymptomatic. The patient’s clinical timeline and diagnostic workup are summarized in Table 2.

**Table 2:** Clinical timeline and diagnostic workup.

Time point	Clinical events	Imaging findings	Laboratory/hematologic findings	Interpretation
Day 1	Acute dyspnea, hypoxemia, ECG: S1Q3T3 pattern	CTPA: extensive bilateral PE, RV/LV > 1	D-dimer, troponin, and NT-proBNP elevated	Intermediate-high-risk pulmonary embolism with right ventricular strain
Days 2-6	Clinical stabilization under monitoring	Echocardiography: RV dilation, TAPSE 9 mm	RBC 7.37 × 10⁶/µL, Hb 15 g/dL, Hct 45.9%, MCV 66.5 fL; low ferritin and low transferrin saturation	Suspicion of iron deficiency–masked erythrocytosis
Day 6	Discharge after clinical stabilization	No malignancy on CT; Doppler ultrasonography: no DVT	Low EPO; JAK2 V617F positive; bone marrow biopsy showing panmyelosis and reduced iron stores	Diagnosis of iron deficiency–masked JAK2-positive polycythemia vera
1-mo follow-up	Dyspnea markedly improved	Echocardiographic improvement; TAPSE 19.5 mm	D-dimer and troponin normalized; Hct controlled	Improved right ventricular function with normalization of D-dimer and troponin
3-mo follow-up	Clinically stable, no recurrent thrombotic events	—	—	Stable clinical evolution under hematologic follow-up
1-y follow-up	Asymptomatic	—	Hb 14 g/dL, Hct 42.6%	Stable long-term evolution with sustained hematocrit control

CTPA, computed tomography pulmonary angiography; DVT, deep vein thrombosis; ECG, electrocardiogram; EPO, erythropoietin; Hb, hemoglobin; Hct, hematocrit; MCV, mean corpuscular volume; PE, pulmonary embolism; RBC, red blood cells; RV/LV, right ventricular-to-left ventricular; TAPSE, tricuspid annular plane systolic excursion.

## Discussion

Polycythemia vera (PV) is a classical BCR-ABL1–negative myeloproliferative neoplasm (MPN), characterized by clonal erythrocytosis, most commonly driven by the JAK2 V617F mutation, which causes constitutive activation of the JAK-STAT signaling pathway [1,5]. PV belongs to the spectrum of classical MPNs together with essential thrombocythemia (ET), primary myelofibrosis (PMF), and unclassifiable myeloproliferative neoplasms (MPN-U) [6].

The JAK2 V617F mutation, a somatic G-to-T substitution in exon 14, is detected in approximately 95%-96% of PV cases, while a smaller minority have mutations involving exon 12 of JAK2. These mutations are highly characteristic of PV and have significantly improved diagnostic accuracy. Higher JAK2 V617F allele burden, leukocytosis, and increased phlebotomy requirements are associated with a greater thrombotic risk [7]. The allele burden**,** expressed as variant allele frequency, reflects the proportion of hematopoietic cells carrying the mutation and correlates with disease phenotype. It has been associated with thrombotic risk, disease progression and survival. The allele burden is generally lowest in essential thrombocythemia and higher in polycythemia vera and primary myelofibrosis. In PV, a higher allelic burden (above 50%-58%) is linked to more aggressive disease, including leukocyte count, hemoglobin levels, lactate dehydrogenase, splenomegaly, and poorer overall survival [14].

JAK2 V617F allele burden has been associated with disease phenotype and thrombotic risk in myeloproliferative neoplasms. Although higher allele burden is generally linked to a more proliferative phenotype, thrombotic complications may also occur in patients with relatively low allele burden, as illustrated in our case. This observation suggests that thrombogenesis in masked PV is multifactorial and may involve additional contributors such as leukocytosis, inflammation, endothelial dysfunction, and iron deficiency [14,16,18].

Despite these advances, masked polycythemia vera (mPV) remains a diagnostic challenge and an area of increasing clinical interest. The term refers to patients who do not initially present with the overt hemoglobin or hematocrit elevations classically associated with PV, yet exhibit clinical, molecular, and bone marrow features consistent with the disease [8]. Recognition of mPV has increased following revisions to the World Health Organization diagnostic criteria. In 2016, the hemoglobin and hematocrit thresholds were lowered to improve identification of patients with borderline erythrocytosis and atypical presentations, while the 2022 classification further emphasized the significance of masked disease, the confounding effect of iron deficiency, and the contribution of molecular markers to diagnosis and risk stratification [9]. Failure to recognize mPV in a timely manner may expose patients to thrombotic complications and delay initiation of appropriate disease-specific therapy.

In this context, JAK2 testing has become integral to the diagnostic workup, although its specificity is limited by its occurrence in other myeloproliferative neoplasms, mPV may be regarded as a distinct entity along the polycythemia vera–essential thrombocythemia continuum, highlighting the importance of extended molecular assessment, particularly evaluation for JAK2 exon 12 mutations in diagnostically challenging or JAK2 V617F-negative cases. Although isotopic red cell mass measurement is no longer incorporated into WHO diagnostic criteria and has largely fallen out of routine clinical use because of its cumbersome nature, cost, and limited diagnostic performance, it may still provide adjunctive value in selected cases [12].

Importantly, recognition of masked PV has substantial clinical relevance because these patients appear to carry a vascular risk comparable to, and in some studies greater than, that of overt PV. Alvarez-Larrán et al. [11] showed that patients with masked PV had less pronounced erythrocytosis, a lower JAK2 V617F allele burden, and frequently higher platelet counts than those with overt PV, yet their thrombotic risk remained clinically significant. Similarly, Barbui et al. [13] reported a higher thrombotic burden in masked PV, with venous thrombosis observed in 10% versus 6% and arterial thrombosis in 21% versus 10% of patients with overt PV. Collectively, these findings support the view that mPV is not a benign variant of disease but a clinically meaningful presentation of PV that requires the same diagnostic vigilance and therapeutic approach as overt PV, including strict maintenance of hematocrit below 45%. Masked PV refers to a clinicopathologic form of PV in which patients do not meet traditional hemoglobin or hematocrit thresholds but fulfill other diagnostic criteria, including JAK2 mutation and characteristic bone marrow morphology. Pulmonary embolism, though rare, may serve as the presenting manifestation of polycythemia vera (PV), including in patients with masked PV**,** where overt erythrocytosis is absent at presentation [11,13,17]. Interestingly, no lower-limb deep vein thrombosis was identified in our patient. This may suggest either an occult venous source, particularly within the pelvic circulation, or the possibility of in situ pulmonary thrombosis in the setting of a persistent prothrombotic state.

This case illustrates a key diagnostic limitation: hemoglobin and hematocrit are concentration-based parameters, and may underestimate erythrocytosis when red cells are markedly microcytic. In such patients, an elevated red blood cell count and iron deficiency should prompt further evaluation for occult PV [11]. Iron deficiency represents a major cause of this diagnostic pitfall, as it reduces both hemoglobin concentration and mean corpuscular volume while masking the underlying increase in red cell mass. Consequently, PV may remain clinically occult until a thrombotic complication arises or molecular investigation is undertaken. This diagnostic challenge is particularly relevant in patients with unexplained microcytosis, elevated red blood cell count, leukocytosis, thrombocytosis, low serum erythropoietin, or thromboembolic events despite nonelevated or high-normal hemoglobin and hematocrit values. In our patient, marked iron deficiency likely concealed the underlying erythrocytosis and contributed to deceptively unremarkable hematologic indices at presentation [12]**.** In this setting, an elevated red blood cell count associated with marked microcytosis becomes an important clue to masked PV [15].

Thrombotic complications remain a major cause of morbidity and mortality in masked PV and may be the first clinical manifestation of the disease, preceding hematologic recognition of the underlying myeloproliferative neoplasm [1]. This appears particularly relevant when erythrocytosis is attenuated by iron deficiency, as illustrated in our patient. Previous studies have suggested that patients with masked PV may experience thrombohemorrhagic complications at rates comparable to, or even higher than, those of patients with overt PV, partly because diagnosis and treatment are delayed [10].

Given that the underlying clonal disorder is a chronic and persistent prothrombotic condition, it may often warrant prolonged antithrombotic management following a thrombotic event. Thromboembolic and cardiovascular complications are common causes of morbidity and mortality. In PV, thrombotic risk is particularly high around diagnosis and tends to decrease with effective disease control. Established risk factors include age ≥60 years and prior thrombosis, while elevated hematocrit and leukocytosis further contribute to vascular risk. Accordingly, therapy primarily aims to prevent thrombosis, notably by maintaining hematocrit below 45%, a target associated with reduced cardiovascular death and major thrombotic events. Low-risk patients are generally managed with phlebotomy and low-dose aspirin, whereas high-risk patients require cytoreductive therapy, most commonly hydroxyurea. Interferon and ruxolitinib remain important options in selected patients with intolerance or inadequate response to hydroxyurea [22].

Regarding anticoagulation, available evidence comparing direct oral anticoagulants (DOACs) and vitamin K antagonists (VKAs) in myeloproliferative neoplasms remains limited and largely retrospective. In a cohort of 30 patients with PV or ET and thrombosis, no significant differences were found between DOACs and VKAs regarding recurrent thrombosis or bleeding [19]. A larger retrospective series of 53 MPN patients showed similar results, although bleeding events occurred earlier in those receiving DOACs [20]. Current guidelines do not clearly favor 1 anticoagulant class over the other; while VKAs remain supported by the longest clinical experience, the use of DOACs is increasing despite the scarcity of robust MPN-specific data [21].

For radiologists, extensive or unprovoked pulmonary embolism should prompt careful review of available hematologic parameters. An elevated red blood cell count with marked microcytosis, despite nonelevated hemoglobin or hematocrit levels, should raise suspicion for iron deficiency–masked polycythemia vera and prompt consideration of JAK2 mutation testing.

Only a limited number of reports have described pulmonary embolism as the presenting manifestation of masked polycythemia vera, and even fewer have highlighted the masking effect of iron deficiency on hemoglobin and hematocrit values. The present case is clinically relevant because it illustrates how reliance on conventional erythrocytosis thresholds alone may delay recognition of PV until a major thrombotic event occurs. Careful attention to red blood cell count, microcytosis, and iron studies may help identify occult disease earlier. Therefore, clinicians should consider JAK2 testing in cases of unprovoked venous thromboembolism when subtle hematologic abnormalities such as microcytosis, elevated red blood cell count, leukocytosis, or subnormal erythropoietin are present, even in the absence of overt erythrocytosis. Early molecular testing and bone marrow evaluation are crucial for timely diagnosis and initiation of treatment to reduce thrombotic recurrence and limit disease progression.

## Conclusion

This case illustrates how polycythemia vera can be concealed by severe iron deficiency, resulting in normal or high-normal hemoglobin and hematocrit levels despite underlying clonal erythropoiesis. In patients presenting with unprovoked pulmonary embolism associated with microcytosis and elevated red blood cell count, an occult JAK2-positive myeloproliferative neoplasm should be strongly considered. CTPA and echocardiography played a central role in assessing thrombotic burden, evaluating right heart strain, and disease severity. Early recognition of masked PV is essential for timely initiation of cytoreductive and antithrombotic therapies, reducing the risk of recurrent thrombosis and disease progression.

## Data availability

Data sharing is not applicable to this article as no datasets were generated or analyzed during the current study.

## Ethical approval

Ethics approval was not obtained because this manuscript is a case report.

## Author contributions

All authors contributed to the conception, drafting, critical revision, and final approval of the manuscript.

## Declaration of generative AI and AI-assisted technologies in the writing process

Generative AI tools were used solely to improve language clarity and correct grammatical errors. The authors reviewed and approved all AI-assisted changes and take full responsibility for the content of the manuscript.

## Patient consent

A written informed consent was obtained from the patient for publication of this case report and accompanying images.

## References

[1] Tefferi A., Barbui T. (2023). Polycythemia vera: 2024 update on diagnosis, risk-stratification, and management. Am J Hematol.

[2] Arber D.A., Orazi A., Hasserjian R.P., Borowitz M.J., Calvo K.R., Kvasnicka H.M. (2022). International consensus classification of myeloid neoplasms and acute leukemias: integrating morphologic, clinical, and genomic data. Blood.

[3] Barbui T., Thiele J., Vannucchi A.M., Tefferi A. (2015). Rationale for revision and proposed changes of the WHO diagnostic criteria for polycythemia vera, essential thrombocythemia and primary myelofibrosis. Blood Cancer J.

[4] Huang P., Li Y. (2022). Polycythemia vera presenting with pulmonary embolism and splenic infarction: a case report. J Int Med Res.

[5] McMullin M.F., Harrison C.N., Ali S., Cargo C., Chen F., Ewing J. (2019). A guideline for the diagnosis and management of polycythaemia vera: a British Society for Haematology guideline. Br J Haematol.

[6] Thiele J., Kvasnicka H.M., Orazi A., Gianelli U., Gangat N., Vannucchi A.M. (2023). The international consensus classification of myeloid neoplasms and acute leukemias: myeloproliferative neoplasms. Am J Hematol.

[7] Iurlo A., Cattaneo D., Bucelli C., Baldini L. (2020). New perspectives on polycythemia vera: from diagnosis to therapy. Int J Mol Sci.

[8] Tefferi A. (2016). Myeloproliferative neoplasms: a decade of discoveries and treatment advances. Am J Hematol.

[9] Durak Z.A., Demirci Z., Eşkazan A.E. (2020). Masked polycythemia vera: diagnostic challenges and clinical implications. Leuk Res.

[10] Lussana F., Carobbio A., Randi M.L., Elena C., Rumi E., Finazzi G. (2014). A lower intensity of treatment may underlie the increased risk of thrombosis in young patients with masked polycythaemia vera. Br J Haematol.

[11] Alvarez-Larrán A., Angona A., Ancochea A., García-Pallarols F., Fernández C., Longarón R. (2016). Masked polycythaemia vera: presenting features, response to treatment, and clinical outcomes. Am J Hematol.

[12] Durak Z.A., Demirci Z., Eşkazan A.E. (2026). Masked polycythemia vera: diagnostic challenges and clinical implications. Expert Rev Hematol.

[13] Barbui T., Thiele J., Gisslinger H., Finazzi G., Carobbio A., Rumi E. (2014). Masked polycythemia vera: results of an international study. Am J Hematol.

[14] Lee A.J., Kim S.G., Nam J.Y., Yun J., Ryoo H.M., Bae S.H. (2021). Clinical features and outcomes of JAK2 V617F-positive polycythemia vera and essential thrombocythemia according to the JAK2 V617F allele burden. Blood Res.

[15] Almeida L.R., Faustino D., Gameiro R., Salvado V., Dias L. (2023). Masked polycythemia vera and iron deficiency in a fertile-age woman. Cureus.

[16] Jimenez K., Leitner F., Leitner A., Scharbert G., Schwabl P., Kramer A.M. (2021). Iron deficiency-induced thrombocytosis increases thrombotic tendency in rats. Haematologica.

[17] Barbui T, Carobbio A, Rumi E, Finazzi G, Gisslinger H, Rodeghiero F (2014). In contemporary patients with polycythemia vera, rates of thrombosis and risk factors delineate a new clinical epidemiology. Blood.

[18] Nashashibi J., Avraham G.R., Schwartz N., Awni Y., Elias M. (2019). Intravenous iron treatment reduces coagulability in patients with iron deficiency anaemia: a longitudinal study. Br J Haematol.

[19] Fedorov K., Goel S., Kushnir M., Billett H. (2021). Thrombosis in myeloproliferative neoplasms: treatment outcomes of direct oral anticoagulants and vitamin K antagonists. Res Pract Thromb Haemost.

[20] Fedorov K., Goel S., Kushnir M., Billett H.H. (2019). Direct oral anticoagulants for prevention of recurrent thrombosis in myeloproliferative neoplasms. Blood.

[21] How J., Story C., Connors J.M. (2021). Prevention of recurrent thromboembolism in myeloproliferative neoplasms: review of literature and focus on direct oral anticoagulants. Postgrad Med.

[22] Griesshammer M., Kiladjian J.J., Besses C. (2019). Thromboembolic events in polycythemia vera. Ann Hematol.

